# Clinical Determinants of Disease Progression in Patients With Beta-Sarcoglycan Gene Mutations

**DOI:** 10.3389/fneur.2021.657949

**Published:** 2021-07-01

**Authors:** Giulia Bruna Marchetti, Luca Valenti, Yvan Torrente

**Affiliations:** ^1^Unit of Neurology, Department of Pathophysiology and Transplantation, Università degli Studi di Milano, Fondazione Istituto di Ricerca e Cura a Carattere Scientifico Cà Granda Ospedale Maggiore Policlinico, Centro Dino Ferrari, Milan, Italy; ^2^Department of Pathophysiology and Transplantation, Department of Transfusion Medicine and Hematology, Translational Medicine, Università degli Studi di Milano, Fondazione Istituto di Ricerca e Cura a Carattere Scientifico Ca' Granda, Milan, Italy

**Keywords:** limb-girdle muscular dystrophy, type 2E, creatine kinase, respiratory function tests, echocardiography, disease progression

## Abstract

**Background:** Limb-girdle muscular dystrophy 2E (LGMD 2E), recently renamed as autosomal recessive limb-girdle muscular dystrophy-4 (LGMDR4), is characterized by the lack of beta-sarcoglycan, normally expressed in skeletal muscles and cardiomyocytes. We hypothesized that progressive respiratory and left ventricular (LV) failure in LGMDR4 could be associated with the age and interrelated phenomena of the disease's natural history.

**Methods:** We conducted a retrospective review of the records of 26 patients with LGMDR4. Our primary objective was to compare the rates of decline among creatine phosphokinase (CPK) values, pulmonary function test (PFT) measures, and echocardiographic estimates and to relate them to patients' age.

**Results:** The rates of decline/year of CPK, PFTs, and LV function estimates are significatively bound to age, with the LV ejection fraction (EF) being the strongest independent variable describing disease progression. Moreover, the rate of decline of CPK, PFTs, and LV differed in patients grouped according to their genetic mutations, demonstrating a possible genotype–phenotype correlation. The parallel trend of decline in CPK, PFT, and EF values demonstrates the presence in LGMDR4 of a simultaneous and progressive deterioration in muscular, respiratory, and cardiac function.

**Conclusions:** This study expands the current knowledge regarding the trend of CPK values and cardiac and respiratory impairment in patients with LGMDR4, to optimize the monitoring of these patients, to improve their quality of life, and to provide clinical indices capable of quantifying the effects of any new gene or drug therapy.

## Background

Limb-girdle muscular dystrophy 2E (LGMD 2E), recently reclassified as limb-girdle muscular dystrophy recessive type 4 (LGMDR4) (1), is a rare type of recessive muscular dystrophy caused by the lack of beta-sarcoglycan. Sarcoglycans (SGs) count four transmembrane proteins: alpha (a-), beta (b-), gamma (c-), and delta-sarcoglycan (d-SG), organized in a tetramer that stabilizes the dystrophin-associated glycoprotein complex (2). These proteins are expressed in the sarcolemma of smooth and striated muscular fibers. Bi-allelic mutations in the genes of each sarcoglycan cause specific subtypes of recessive LGMD: mutations on the a-SG gene (SGCA) lead to LGMDR3, on the b-SG gene (SGCB) to LGMDR4, and on the c-SG gene (SGCC) to LGMDR5 (3), while mutations on the d-SG gene have been discovered to cause the extremely rare LGMDR6 (4). The SGs assemble during a stepwise process, which mainly depends on b-SG, which initiates the assembly and whose association with d-SG is fundamental for the correct localization of the whole complex in the membrane (5). Different roles of SGs partially explain the wide clinical differences among sarcoglycanopathies: age of onset, disease progression, muscle, joint, cardiac, and respiratory involvement can vary greatly. Because of its fundamental role, b-SG's absence causes a severe form of LGMD, which debuts with a progressive weakening of proximal muscles in early childhood (6). The presence of respiratory and heart dysfunction makes LGMDR4 close to Duchenne muscular dystrophy, although the pathophysiology of cardiac anomalies might be different (7). More than 60% of LGMDR4 patients suffer from dilated cardiomyopathy (8), a condition that is less frequently observed in LGMDR3 and LGMDR5 (9). Also, LGMDR4 is often associated with respiratory failure (10), a leading cause of death in affected individuals that is nowadays successfully prevented using non-invasive ventilation (NIV) (11). Aside from this little information, the natural history of LGMDR4 is still poorly known because of its rareness (prevalence is estimated around 0.86 × 10^−6^ individuals) and of the great difficulties in collecting data from so few and scattered patients. In literature, studies on LGMDR4 patients mainly consider outcome measures based on motor function of the lower and upper limbs (12). Recently Alonso-Pérez et al. (13) established that early onset and low protein expression in muscle biopsy (<30%) are independent risk factors for loss of ambulation before 18 years of age in sarcoglycanopathies. Nonetheless, the evaluation of lung function and respiratory muscle activity during disease progression should prevent ventilatory insufficiency improving the quality of life and could be a marker for new gene-modifying and pharmacological therapeutic strategies (14, 15). However, despite broad acceptance of the prognostic significance of cardiac and respiratory involvement in sarcoglycanopathies (7, 10, 16, 17), there remains little knowledge on the course of these symptoms over natural history of disease. The present study was designed with the aim to improve the management and long-term outcome of LGMDR4 patients.

## Materials and Methods

### Aims

In this study, we investigated the possible association between progressive respiratory and LV failure and the age in LGMDR4 and their connection to interrelated phenomena of the disease's natural history. Specific aims were to study the evolution of CPK levels and cardiac and respiratory functions in terms of spirometry and echocardiography pattern from childhood to adulthood, to identify possible key points in the natural course of the disease, and to improve the given treatments. Furthermore, we evaluated the strength of a possible genotype–phenotype correlation.

### Design

This study was designed as a 10-year observational retrospective study, collecting clinical data from 26 patients with a genetic diagnosis of LGMDR4. To gain a better knowledge of the natural history of this uncommon condition, we highlighted the significative correlation between CPK and cardiorespiratory assessments and disease progression over years. The interconnection between variables was evaluated in all patients and after stratification by presence or absence of a common genetic mutation in exon 3, associated with a severe phenotype (4, 18).

### Patients

Twenty-six LGMDR4 patients, with a genetically defined diagnosis, were enrolled, ranging in age from 8 to 55 years and with data collected on at least three different visits. Patients were enrolled within the “Gruppo Familiari Beta-sarcoglicanopatie” Italian network and evaluated in different Italian centers once or twice per year, for a total of 542 visits (which include 216 neurological, 177 cardiological, and 149 pneumological examinations). All patients or parents signed a consent form, approved by the local ethics committee according to the declaration of Helsinki. The study was approved by the Ethics Committee of the Fondazione IRCCS Ca' Granda Ospedale Policlinico of Milan (EC approval 386_2020, May 19, 2020) and registered on ClinicalTrials.gov with the following number: NCT04509609.

### Clinical Tests

All clinical data were provided by patients, on paper or by informatic support. Data were collected in a table, purposely created. We recorded the genetic, histological, and immunohistochemical tests that led to diagnosis as well as related information such as age at onset and/or at diagnosis, first symptoms, and family history. Every neurological, cardiological, or pulmonological examination performed over the years was reported. Functional, blood, and laboratory tests were included as well. Each data that could help determine the disease's evolution was collected. If present, we reported any sign defining muscular involvement (hypotrophy, pseudohypertrophy, weakening grade and distribution, ability to stand on toe, dysphagia, scapular winging, spinal deformity, and so on) and any kind of muscular function assessment done over the years. Often patients followed in different hospitals underwent different evaluations, which were all collected on the off chance to determine which best describes muscle impairment. Respiratory dysfunction was assessed by spirometry and overnight oximetry. Pulmonary function test variables (expressed as percentage of predicted) consisting of vital capacity (VC), forced vital capacity (FVC), and forced expiratory volume in first second (FEV1) were included in the study analyses. Also, we reported ventilatory and cough parameters: maximal voluntary ventilation (MVV), maximal inspiratory pressure (MIP), and maximal expiratory pressure (MEP). Ejection fraction (EF) data were collected from the echocardiogram reports.

### Statistical Analysis

**Figure 1:**
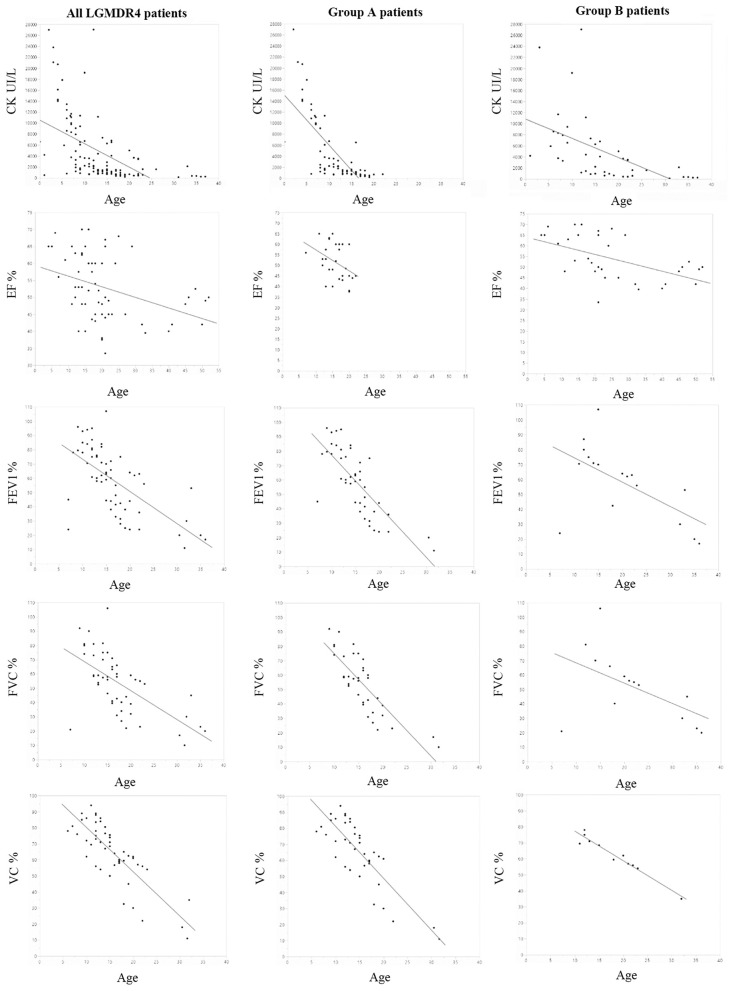
All graphics were created using JMP by SAS. First line: evolution of CPK in all patients and in groups A and B over the years: on the Y axis, CPK values expressed in UI/L; on the X axis, age expressed in years (*p*-value 8.3e-10 total patients, *p* = 2E-13 group A and *p* = 2.80E-04 group B). Second line: evolution of EF in all patients and in groups A and B over the years: on the Y axis, EF values expressed as percentage; on the X axis, age expressed in years (*p*-value 0.0023 total patients, *p* = 3.40E-02 group A and *p* = 1.2E-3 group B). Third line: evolution of FEV1 in all patients and in groups A and B over the years: on the Y axis, FEV1 values expressed as percentage; on the X axis, age expressed in years (*p*-value 5.96e-9 total patients, *p* = 3E-10 group A and *p* =0.01 group B). Fourth line: evolution of FVC in all patients and in groups A and B over the years: on the Y axis, FVC values expressed as percentage of predicted value; on the X axis, age expressed in years (*p*-value 2.42e-6 total patients, *p* = 5.01E-10 group A and *p* =0.20 group B). Fifth line: evolution of VC in all patients and in groups A and B over the years: on the Y axis, VC values expressed as percentage of predicted value; on the X axis, age expressed in years (*p* = 0.0002 total patients, *p* = 2.1E-3 group A and *p* = 5.7E-7 group B).

**Figure 2:**
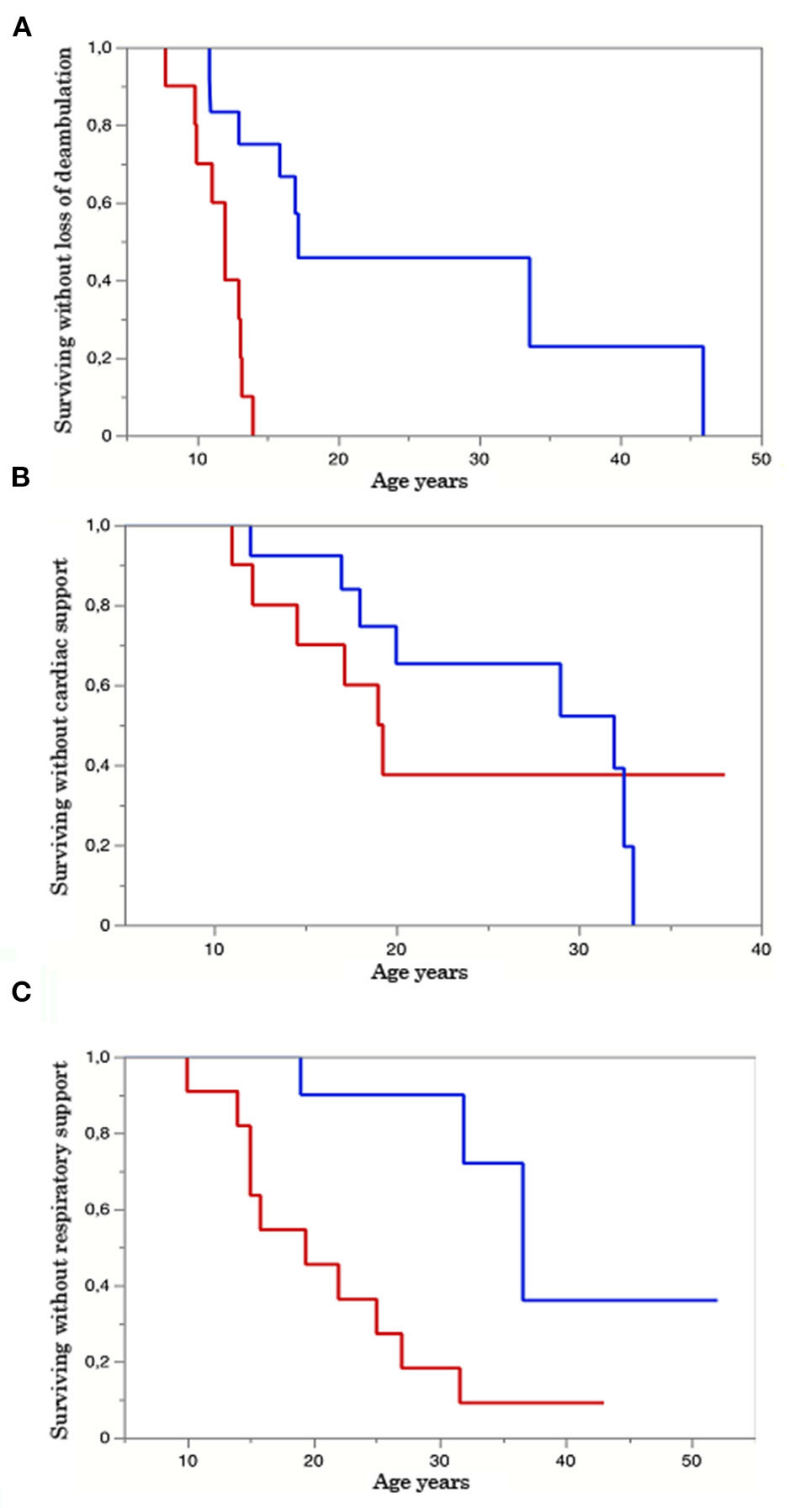
Kaplan–Meyer (KM) survival curve. Group A red line, group B blue line. The Y axis represents the percentage of survival, and the X axis shows the age in years. **(A)** KM curves of patients surviving without loss of ambulation in both groups A and B. *p*-value = 0.0005. **(B)** KM curves of patients surviving without assuming cardiological therapy in groups A and B. *p*-value = 0.14. **(C)**. KM curves of patients surviving without respiratory aids in groups A and B. *p*-value = 0.0018.

We ordered each variable available according to patients' age. Not for every variable and not for every patient we obtain enough data to have a significant description of the disease's natural history. Otherwise, for some variables we were able to describe a lifetime trend. As it has been recently demonstrated that patients with truncating mutations, and therefore lower proteins' concentrations, show a severer clinical progression (15), and in view of our preliminary observations, we tried to highlight any genotype-phenotype correlation. On this purpose, patients were analyzed both as total and as grouped in genetic subgroups: group A, for patients carrying in homozygosis or in compound heterozygosis a specific truncating mutation in exon 3 (c.377_384duplCAGTAGGA), known to cause severe disease (4, 18), and group B for patients with every other kind of alteration. In our cluster, variants were found in exons 3, 4, 6, and 1, which are known for causing a mild disease (18). Group A included 12 patients (6 men and 6 women, median age 25 years) and group B 14 (7 men and 7 women, median age 32). At first, we ordered data according to age, for the overall cohort and for the genetic subgroups. When multiple measurements were available for an individual's age, the mean adjusted value was used. This enabled us to improve data samples for each age and to compare disease progression between groups. Our first aim was to examine the trend of clinical variables over the years, both in the overall cohort and in patients stratified by genotype. We first analyzed the correlation between clinical measures and disease progression over the years, using age as the independent variable (Figure 1). Hence, we evaluated whether the trend of cardiac and respiratory function over the years was independent of CPK considering age as the dependent variable which affect the progression of disease (Tables 1–3). Finally, we made an exploratory analysis to investigate the correlation between clinical variables and clinical outcomes, defined as a loss in ambulatory, cardiac and respiratory function. For this analysis, age at disability was used as the dependent variable (Supplementary Table 1). Analyses were performed using generalized linear models: linear regression models were fit to analyze continuous traits, with dependent variables such as CPK, EF, VC, FVC, and FEV1. To assess the major independent predictors of clinically relevant outcomes, models were adjusted for confounding factors, as specified in section Results. Variables with skewed distributions were logarithmically transformed before to enter the models. For all the analyses, we considered every data available and grouped them for age of observation, independently from patient identity. We conducted time to event analyses for the time from disease onset to loss of specific functions related to motor/cardiac/respiratory assessments. In order to reduce the uncertainties that reflect the lower number of patients and variability in their follow-up (Tables 4, 5), we considered as objective disability events the (i) age of loss of ambulation, (ii) age of introduction of cardiac medications, and (iii) age of introduction of respiratory support. Time to loss of ambulation, cardiological therapy, and respiratory aids were calculated using a Kaplan–Meyer (KM) plot and log-rank tests (Figure 2). The survival curve was carried out using JMP 12.0 (SAS Institute, Cary, NC, USA), and *P*-values <0.05 were considered statistically significant.

**Table 1:** Evolution in clinical variables with ageing in LGMDR4.

	**Univariate**			**Multivariate model 1**			**Multivariate model 2**			**Multivariate model 3**		
	**Estimate**	**Std error**	***P*-value**	**Estimate**	**Std error**	***P*-value**	**Estimate**	**Std error**	***P*-value**	**Estimate**	**Std error**	***P*-value**
CPK, IU/L (*n* = 114)	−6.59E-04	9.87E-05	8.30E-10^*^	−4.15E-5	4.39E-04	0.92	−7.71E-04	4.31E-04	0.079	2.18E-04	5.77E-04	0.71
EF, % (*n* = 62)	−4.48E-01	1.41E-01	0.0023^*^	−0.27E-01	7.89E-02	0.0019^*^				−2.80E-01	1.13E-01	0.0201^*^
VC, % (*n* = 52)	−7.45E-02	1.89E-02	0.0002^*^									
FVC, % (*n* = 51)	−1.75E-01	3.31E-02	2.42E-06^*^									
FEV1, % (*n* = 62)	−1.86E-01	2.77E-02	5.96E-09^*^				−1.28E-01	3.92E-02	0.0021^*^	−5.30E-01	5.45E-01	0.34

*CPK, Creatine Phospokinase; EF, ejection fraction; VC, Vital Capacity, considered as percentage of predicted; FVC, Forced Vital Capacity, considered as percentage of predicted; FEV1, Forced Expiratory Volume in the first second considered as percentage of predicted*.

*N = number of observations recorded*.

*Multivariate linear regression models (three alternative models, considering different combinations of variables) were considered to examine more robust independent biomarkers of disease progression. Multivariate model 1 was adjusted for 42 observations, multivariate model 2 was adjusted for 25 observations, and multivariate model 3 was adjusted for 21 observations*.

* *Stands statistically significative values*.

**Table 2:** Evolution of clinical variables with aging in LGMDR4 groups A and B, as estimated by univariate generalized linear regression.

	**Univariate group A**					**Univariate group B**				
	***N***	**Line regression**	**Estimate**	**Std error**	***P*-value**	***N***	**Line regression**	**Estimate**	**Std error**	***P*-value**
CPK, IU/L	70	15,069.72–907.52	−9.08E+02	9.97E+01	2E-13^*^	43	10,770.49–344.96	−3.45E+02	8.69E+01	2.80E-04^*^
EF, %	28	67.28–0.99	−0.99	0.44	3.40E-02^*^	34	63.84–0.39	−0.39	0.11	1.2E-3^*^
VC, %	41	124.03–3.56	−3.56	1.08	2.1E-3^*^	11	95.75–1.85	−1.85	0.15	5.7E-7^*^
FVC, %	37	110.49–3.56	−3.56	0.42	5.01E-10^*^	14	71.49–1.12	−1.12	0.82	0.20
FEV1, %	45	112.76–3.55	−3.55	0.44	3E-10^*^	17	90.98–1.64	−1.64	0.57	0.01^*^

*CPK, Creatine Phosphokinase; EF, ejection fraction; VC, Vital Capacity, considered as percentage of predicted; FVC, Forced Vital Capacity, considered as percentage of predicted; FEV1, Forced Expiratory Volume in the first second considered as percentage of predicted*.

*N = number of observations recorded*.

* *Stands statistically significative values*.

**Table 3:** Correlation between variables.

**Variable 1**	**Variable 2**	**Correlation**	***N***	***P*-value**
CPK, UI/L	Age	−0.53	114	1.30E-09^*^
EF, %	Age	−0.37	62	2.80E-03^*^
EF, %	CPK, UI/L	0.11	25	5.90E-01
VC, %	Age	−0.48	52	3.20E-04^*^
VC, %	CPK, UI/L	0.35	35	4.10E-02^*^
VC, %	EF, %	0.56	25	3.40E-03^*^
FVC, %	Age	−0.59	51	4.20E-06^*^
FVC, %	CPK, UI/L	0.47	36	3.80E-03^*^
FVC, %	EF, %	0.62	28	4.10E-04^*^
FVC, %	VC, %	0.36	29	5.80E-02
FEV1, %	Age	−0.65	62	1.20E-08^*^
FEV1, %	CPK, UI/L	0.52	42	4.70E-04^*^
FEV1, %	EF, %	0.49	30	5.90E-03^*^
FEV1, %	VC, %	0.42	39	8.60E-03^*^
FEV1, %	FVC, %	0.95	48	7.00E-26^*^

*CPK, Creatine Phosphokinase; EF, ejection fraction; VC, Vital Capacity, considered as percentage of predicted; FVC, Forced Vital Capacity, considered as percentage of predicted; FEV1, Forced Expiratory Volume in the first second considered as percentage of predicted*.

*N = number of observations recorded*.

*Each variable was related to all other variables under study*.

* *Stands statistically significative values*.

**Table 4:** Demographic and clinical features of the studied cohort.

**ID**	**Gr**.	**Onset/sex**	**L.E**.	**Walk/A.L**.	**Steroid/age**	**CM**	**Heart A.S**.	**LRD**	**Lungs A.S**.	**Scol**.	**N1 6 MW/Y**	**L. 6 MW/Y**	**LI**	**N1 10 mW/Y**	**L. 10 mW/Y**	**LI**	**N1 MFM/Y**	**L. MFM/Y**	**LI**	**N1 NSAA/Y**	**L. NSAA/Y**	**LI**	**N1 WGM/Y**	**L. WGM/Y**	**LI**
1	A	7/M	16	N/11	Y 7–10 y	Y	12	Y	15	Y	366/7 y	341/10 y	8.3	7.5/7 y	25.56/11 y	−4.5	//	//	//	25/7 y	0/15 y	3.13	//	//	//
2	A	5/M	20	N/13	Y 10–17 y	Y	17	Y	19	Y	235/10 y	124/12 y	55.5	7/9y	17.75/16 y	−1.5	//	//	//	8/10 y	9/11 y	−1.00	//	//	//
3	A	5/M	22	N/13	Y 8–11 y	Y	15	Y	15	Y	//	//	//	8/8 y	//	//	28.13/15 y	15.63/20 y	2.5	//	//	//	9/16 y	//	//
4	A	4/F	19	N/10	N	Y	19	Y	16	Y	//	//	//	6/7 y	//	//	50/9 y	17.71/19 y	3.2	//	//	//	$\frac{1}{4}$ y	9/13 y	−0.78
5	B	10/M	33	Y	N	N	33	N	N	N	260/33 y	//	//	//	//	//	//	//	//	//	//	//	//	//	//
6	A	2/F	19	N/13	N	N	N	Y	14	Y	//	//	//	//	//	//	//	//	//	//	//	//	2/2 y	4/10 y	−0.25
7	A	3/F	18	N/8	N	N	19	Y	10	Y	//	//	//	//	//	//	//	//	//	//	//	//	//	//	//
8	B	2/F	15	N/11	N	N	12	Y	N	Y	301/10 y	//	//	8.5/10 y	//	//	//	//	//	21/10 y	//	//	3/10 y	//	//
9	B	19/M	52	N/46	N	Y	29	N	N	N	//	//	//	//	//	//	//	//	//	//	//	//	//	//	//
10	B	5/F	37	N/17	N	Y	32	Y	37	Y	//	//	//	//	//	//	//	//	//	//	//	//	9/32y	//	//
11	B	6/F	23	N/16	Y 13 y	N	18	Y	19	Y	//	//	//	//	//	//	//	//	//	//	//	//	5/15y	//	//
12	B	26/F	28	Y	N	N	N	N	N	N	//	//	//	//	//	//	//	//	//	//	//	//	0/20 y	//	//
13	A	1.5/M	14	N/12	//	Y	//	//	//	//	//	//	//	//	//	//	//	//	//	//	//	//	//	//	//
14	B	7/M	20	N/11	//	Y	20	N	N	Y	//	//	//	//	//	//	//	//	//	//	//	//	//	//	//
15	A	11/M	43	N///	//	N	N	Y	//	//	//	//	//	//	//	//	//	//	//	//	//	//	//	9/43 y	//
16	A	3/F	34	N/10	//	N	N	Y	27	Y	//	//	//	//	//	//	//	//	//	//	//	//	8/10 y	//	//
17	B	7/M	19	N/17	//	Y	17	N	N	Y	//	//	//	//	//	//	//	//	//	//	//	//	//	//	//
18	A	4/F	20	N/14	Y 25 y	Y	11	Y	25	Y	//	//	//	//	//	//	//	//	//	//	//	//	1/4 y	2/18 y	−0.07
19	B	12/M	16	Y	//	N	N	N	N	N	//	//	//	//	//	//	//	//	//	//	//	//	1/12 y	1/14 y	0.00
20	A	15/M	27	N///	//	Y	//	N	N	N	//	//	//	//	//	//	//	//	//	//	//	//	//	//	//
21	B	1/F	32	//	//	N	N	Y	//	//	//	//	//	//	//	//	//	//	//	//	//	//	//	//	//
22	B	7/F	17	Y	//	N	N	N	N	N	//	//	//	//	//	//	//	//	//	//	//	//	//	//	//
23	B	9/F	21	Y	//	N	N	N	//	N	//	//	//	//	//	//	//	//	//	//	//	//	//	//	//
24	A	2/F	38	N/12	N	N	N	Y	32	N	//	//	//	//	//	//	//	//	//	//	//	//	5/10 y	8/13 y	−1.00
25	B	5/M	32	N/13	N	N	32	Y	32	Y	//	//	//	//	//	//	//	//	//	//	//	//	//	//	//
26	B	4/M	7	Y	N	N	N	N	N	N	345/4 y	480/7 y	−45	4.9/4 y	//	//	//	//	//	25/4 y	//	//	//	//	//

*Y stands for yes, N for no. Empty cells mean no value was available. Columns titles: ID, patient identity; Gr., genotypic group; Onset/sex, age at onset/sex; L.E., age at last evaluation; Walk/A.L., able to walk/age at loss; Steroid/age, steroid therapy assumed/age at assumption; CM, cardiomyopathy; Heart A.S., heart: age at introduction cardiac support therapy; LRD, lungs restrictive deficit; Lungs A.S., lung: age at introduction respiratory support therapy; Scol., scoliosis; N1 6MWT/Y, first 6 min walking test performed and age in years; L. 6MWT/Y, last 6 min walking test performed and age in years; N1 10 mW/Y, first 10 meter walk performed and age in years; L.10 mtW/Y, last 10 meter walk /age in years; N1 MFM/Y, first Muscular function measurement performed and age in years; L. MFM/Y, last Muscular function measurement and age in years; N1 NSAA/Y, first North Star Ambulatory Assessment performed and age in years; L. NSAA/Y, last North Star Ambulatory Assessment and age in years; N1 WGM/Y, first Walton and Gardner-Medwin scale available and age in years; L. WGM/Y, last Walton and Gardner-Medwin scale age in years; LI, annual loss index calculated as first value-last value divided for years passed, always referred to previous test*.

* *stands statistically significative values*.

**Table 5:** MRI and surgical data of the studied cohort.

**Patient ID**	**Gr**.	**Onset/sex**	**First MRI/age**	**Last MRI/age**	**Surgeries**
1	A	7/M	Lower limbs with partial fatty replacement and oedema/9 Y	Lower limbs with hypotrophy, complete fatty replacement of proximal posterior muscles and oedema. Upper limbs with partial fatty replacement/11 y	At 17 y Implantable cardioverter defibrillator (ICD) insertion
2	A	5/M	Upper limbs with almost complete fatty replacement and oedema/14 y	//	//
3	A	5/M	Lower limbs with atrophy and fatty replacement/17 y	//	At 13 y bilateral achilles tenotomy
4	A	4/F	//	//	At 11 y bilateral achilles tenotomy
5	B	10/M	//		//
6	A	2/F	Lower limbs with slight fatty replacement/7 y	//	At 13 y bilateral achilles tenotomy
7	A	3/F	//	//	At 11 y right achilles tenotomy, right knee hemiepiphysiodesis and right tarsal resection. At 13 y vertebral arthrodesis
8	B	2/F	Lower limbs with complete fatty replacement and oedema. Milder involvement in upper limbs/15 y	//	At 15 y bilateral achilles tenotomy
9	B	19/M	//	//	//
10	B	5/F	Proximal upper and lower limbs with atrophy, fatty replacement, and degeneration/4 y	Complete muscle atrophy of upper limbs/20 y	At 14 y bilateral achilles tenotomy
11	B	6/F	Proximal upper and lower limbs with atrophy, fatty replacement and degeneration/13 y	//	//
12	B	26/F	//	//	//
13	A	1.5/M	//	//	//
14	B	7/M	//	//	//
15	A	11/M	Upper limbs with complete fibro-fatty replacement/40 y	//	//
16	A	3/F	//	//	//
17	B	7/M	//	//	//
18	A	4/F	//	//	At 25 y heart transplant
19	B	12/M	//	//	//
20	A	15/M	//	//	//
21	B	1/F	//	//	//
22	B	7/F	Proximal upper and lower limbs with atrophy, fatty replacement, and degeneration/18 y	//	//
23	B	9/F	Proximal upper and lower limbs with atrophy, fatty replacement and degeneration/27 y	//	//
24	A	2/F	//	//	//
25	B	5/M	//	//	//
26	B	4/M	//	//	//

*Empty cells mean no value was available. Columns titles: ID, patient identity; Gr., genotypic group; Onset/ sex, age at onset/sex; First MRI/age, age at first muscle MRI; Last MRI/age, age at last muscle MRI; Surgeries, any surgical procedure reported in clinical charts*.

* *stands statistically significative values*.

## Results

Twenty-six patients (age range 8–55) were included in the study. Each year, each patient underwent on average 1.2 neurological visits, 1.5 cardiological visits, 1.3 pneumological visits, and 5.1 laboratory tests. The mean number of evaluations was 14 for neurological visits (range 3–30), 11.8 for cardiological ones (range 3–16), 11.5 for pneumological ones (range 3–32), and 25.3 for laboratory tests (range, 2–112). The median time interval between the first and last visits was 2.8 years in patients with more than one evaluation. For patients who underwent multiple visits, the mean time between the first and last visits was 15.2 years for neurological assessments (range 2–30), 14.5 for cardiological ones (range 1–37), 12.5 for pneumological ones (range 2–27), and 16.7 for laboratory and imaging tests (range 1–41). Nine patients provided muscle MRI imaging, and only two out of nine have repeated the test twice. No cardiac MRI was available. Motor function measurements were not included in our analysis due to differences in scales attempted across clinical centers. Eleven patients were diagnosed with dilated cardiomyopathy (7 in group A and 4 in group B), but 14 were assuming cardiac medications and according to clinical charts only a few individuals reported occasionally cardiological symptoms such as palpitations or fatigue. Fifteen patients out of the overall cohort suffer from a restrictive lung deficit (10 in group A and 5 in group B), and 12 required the use of devices to support respiratory function (10 in group A and 2 in group B). Moreover, five patients received steroids (4 in group A and 1 in group B), 1 patient of group A underwent cardiac transplant, and 18 participants were non-ambulatory (11 in group A and 7 in group B). Tables 4, 5 summarize demographical and clinical data available for participants that enrolled. We observed a consistent and significant decrease in all biomarkers evaluated (CPK, EF, VC, FVC, FEV1) with aging (Figure 1 and Table 2). As a general rule, group A confirmed to have a more severe disease progression and data obtained from this population show a more homogeneous distribution over age than group B (Figures 1, 2).

### Muscle Impairment: CPK Over Years

CPK value assessments showed a significant decreasing trend over the years (*p*-value 8.30E-10; Table 1). This was not surprising since muscle mass decreases as the disease progresses (Figure 1). CPK peak was around 3 years of age, when first muscular symptoms appear; its values decrease after the ages of 11–13, when most patients of our cluster lost ambulation (mean age of ambulation loss is 17, median age is 13, range 8–46). Interestingly, group A patients showed a constant decrease in CPK values (line slope was −907.52; Figure 1 and Table 2) with a significant regression according to age (*p*-value of 2E-13) (Table 2), whereas for patients in group B the CPK values were more stable and the regression was less significant with age (line slope −344.96; *p*-value 2.8E-4) (Table 2), with the only exception of one peak observed at 12 years in one patient (Figure 1).

### Heart Impairment: EF Over Years

The univariate analysis of EF evolution according to age showed a significant decreasing trend in all patients (Figure 1) (*p*-value 0.0023; Table 1). Notably, differences appear between EF's decreasing trends in groups (Figure 1) with a severe decrease in few years for group A patients (line slope −0.99; Table 2). Group B patients also suffered from cardiac impairment, which still followed a slower progression (line slope −0.39; Table 2). In both groups, we observed a significative relation to age (in group A *p*-value = 0.03, in group B *p*-value = 0.001; Table 2). It is important to notice that in group B we collected data from The elder patient that at age 50 still preserved a good LV function (EF 50%) compared to the younger patient in group A, who at age 20 already suffered from a severe cardiac dysfunction (EF 25%). Both patients assume beta-blocker. One patient of group A was heart-transplanted and received corticosteroid therapy, but no functional tests were available after the transplant.

### Respiratory Impairment: PFT Variables Over Years

Spirometry values were considered as percentages of the predicted value according to age, sex, and height. FEV1, FVC, and VC were studied most frequently in participating patients. Since the majority of spirometry tests were done in sitting position, we excluded supine spirometry tests from three patients. FEV1 (Figure 1), FVC (Figure 1), VC (Figure 1) were significantly related to age in the overall cohort analysis (VC *p*-value = 0.0002, FVC *p*-value = 2.42E-06, FEV1% *p*-value = 5.96E-09; Table 1). A significative trend for all these variables was observed in group A (VC *p*-value = 2.1E-3, FVC *p*-value = 5E-10, FEV1% *p*-value = 3E-10; Table 2). In group B, the univariate analysis was statistically significative only for VC (*p*-value = 5.7E-7) and FEV1% (*p*-value = 1.1E-2) (Table 2). Interestingly, VC reduction is remarkably different between mutation groups. Along with all spirometry values, VC decreases more rapidly in group A than in B in the range of age of 6–12 (line slope for group A 3.56, for group B 1.85) (Figure 1), whereas no difference was noticed between groups at 12 (mean value 77.25% in group A, 76.5% in group B). Moreover, VC decreased significantly at age 18 in group A (mean value 43.33% in group A vs. 59.5% in group B) (Figure 1). Similarly, FVC showed a significant lowering rate in group A vs. group B (line slope for group A = 3.56 and for group B = 1.12; Table 2) with a mean value at 12 of 60.44%, for group A and of 81% for group B (Figure 1). Furthermore, also FEV1 showed the same differences between groups described for VC and FVC (mean value at 12 of 68.4% for group A and of 83.5% for group B) and its variation over the years differs between groups in a significant manner (mean value at 22 years of age at 37.67% for group A and 59.5% for group B). These differences were related to the FEV1 line slope of group A and group B (−3.55 in group A and −1.64 in group B) (Table 2). The correlation between studied variables was therefore investigated (Table 3). A significant pairwise correlation in all combinations of variables was found except for two couples: EF and CPK, and FVC and VC (Table 3).

### Paired Variables' Correlation Analyses

We further studied variable connection, both with paired correlation analysis and with multivariate generalized linear models. As mentioned above, the paired correlation analyses preliminarily demonstrated the independence of CPK to EF as, reversing the factors, the result of the univariate analysis did not change. This data was confirmed by multivariate models. These models were created to test which of the considered markers is more strongly associated with disease progression over the years. Our interest was to evaluate whether parameters of cardiac and respiratory function correlate with disease progression independently of CPK, whose decrease could reflect the reduction in muscle mass. We found that both FEV1 (*p* = 0.0021) and EF (*p* = 0.0019) significantly lower with disease progression, regardless of CPK (Tables 1, 2, models 1 and 2). In a subset of 16 patients (9 from group A and 7 from group B), for whom all these variables were available, we found that EF was the strongest independent marker of disease progression (*p* = 0.0201, Table 1, model 3).

### Variable–Clinical Outcome Correlation Analyses

Finally, in an exploratory analysis conducted in a subset of patients for whom at least two observations were available, we tested whether the trend of disease biomarkers over years/disease progression can predict the age of development of clinical events—disability (Supplementary Table 1). Interestingly, we found a significant association between the rate of decrease in CPK and the age of introduction of respiratory support (*p* = 0.0009). A non-significant trend was also observed between FVC and the age of ambulation loss (*p* = 0.078) (Supplementary Table 1). The Kaplan–Meyer (KM) survival curve together with the log-rank test demonstrated a faster rate of decline in the time of loss of ambulation and introduction of respiratory aids in patients of group A (*p* = 0.0005 and *p* = 0.0018, respectively) (Figures 2A,C). No significant difference was observed among groups concerning KM analysis of cardiological support (*p*-value = 0.14) (Figure 2B). All these data highlight the severe disease course of LGMDR4 patients carrying a Truncating mutation on exon 3.

## Discussion

In this study, we reported retrospective data of 26 LGMDR4 patients. We stratified patients according to their genetic diagnosis to evaluate the impact of the genetic background on the disease natural history. Clinical data collected from younger patients mirrored previous published recommendations (19), as they underwent almost yearly follow-up visits. Otherwise, elder patients and those with a milder phenotype dis not strictly make adequate yearly follow-ups, usually reflecting travel-related difficulties in returning to the examination site. Of note, disease progression in the study cohort seems to be more severe than previously reported in sarcoglycanopathies (19). Particularly in group A, the respiratory support is often introduced earlier than age 29 years and usually associated with scoliosis (Tables 4, 5). Although none of the participants provided cardiac MRI reports, the cardiological assessments were done routinely and cardiological treatments have been generally imposed as soon as instrumental evaluations showed a slight heart impairment. As visible in Tables 4, 5, there is inconsistency between patients assuming cardiological treatments and those with overt cardiomyopathy suggesting the effectiveness of preventive cardiac medicine as previously reported (19).

Both rareness of LGMDR4 and differences in the follow-up routine among hospitals cause data heterogeneity, thus limiting the collection of a valuable number of functional tests in our analysis. Still, among clinical variables analyzed, some showed a clear trend over time that could help to describe the natural history of the disease. CPK, EF, and spirometry values (VC, FVC, FEV1) can help to assess the LGMDR4 evolution. Group A patients demonstrated a faster reduction of enzymes than those in group B. Further, although in both groups CPK was significantly related to age, this correlation was much tighter for group A (*p* = 2E-13; Table 3), probably because of their more severe clinical phenotype. In our analysis, we confirmed that CPK predicts muscular damage evolution, highlighting the importance of periodic CPK assays as previously described for other muscular dystrophies (12). Notably, a steeper rate of decrease in CPK is linked to a younger age of need for respiratory support, as a turning point in the natural history of LGMDR4 (Supplementary Table 1).

In line with the CPK trend, the decline of VC, FVC, and FEV1 was consistent with the progressive nature of LGMDR4 and generally with the type of respiratory impairment affecting neuromuscular patients; VC has always been considered the first marker of inspiratory muscle weakness and the lowering of lung compliance in these patients (20), and FVC and FEV1 are known as important markers of neuromuscular disease progression (21). Notably, in our cluster both FVC and FEV1 showed a faster reduction than VC. These data, although unexpected, can be due to the tight connection between FVC and FEV1 and both expiratory and inspiratory accessory muscles, including upper girdle muscles (22), whereas VC is only partly dependent on muscular function as the largest part of inspiratory work is done by rib-cage compliance. Moreover, the absence of correlation between VC and FVC highlights differences between these variables.

Overall, spirometry variables were independent of CPK and can therefore be eligible to monitor disease evolution. Based on these data, FVC decline may accurately predict the age at loss of ambulation (*p* = 7.83E-02) (see Supplementary Materials).

Only for FEV1 we do have enough data prior to 11 years of age to consent us to observe the relative steadiness of this value before this age when most severe patients have lost the ability to walk. Unfortunately, because of the intrinsic difficulties of the test itself, spirometry can hardly be done before 10 years of age. Nevertheless, it might be important to impose this examination to LGMDR4 patients even in early childhood to gain more knowledge about early respiratory impairment and its pathogenic mechanisms. Particular attention might be paid to FEV1 and FVC, which have already proven reliable in children under the age of 8 years (23) and are significantly related to mortality in neuromuscular diseases (24). All spirometry variables showed a greater reduction rate in group A compared to group B (Table 2). In group A, all three of them had a significant relation to age, while in group B only VC and FEV1 were significantly age-related (Table 2). It is important to notice that spirometry seems to be rarely performed by group B patients, probably because of the relative benignity of their condition. The scarceness of data surely affects the reliability of these results. Our results provide insight into the relevance of EF decrease with aging in the studied cohort. Indeed, we observed a 4.5 ± 1.4% decrease per year in patients for whom this information was available, which was larger than the decrease in FEV1 (−1.9 ± 0.3%) and FVC (−1.7 ± 0.3%) per year. Most importantly, the association between EF's decline and the disease's progression was independent of the rate of decrease in CPK and FEV1 (Figure 1 and Table 1). Although further studies are needed, EF could be a good candidate cardiac marker for estimating LGMDR4 progression regardless of therapy and its use should be implemented throughout the patient's disease to predict and prevent heart failure and cardiovascular events. To add to the overall perspective, cardiac involvement has also shown to be independent from muscular phenotype (13). On the other hand, our data emphasize the importance of yearly spirometry follow-up to measure FVC as predictor of the respiratory insufficiency that can be treated by non-invasive intermittent positive-pressure ventilation with nasal masks.

Concerning the impact of the genetic background, patients of group A have at least one truncating mutation which has already been demonstrated to relate to a more severe phenotype (13). In addition, patients in group A lost ambulation in the early teens or even before, anticipated introduction of respiratory aids, and showed a homogenous decline in CPK, EF, VC, FVC, and FEV1. Moreover, dilated cardiomyopathy is mainly observed in group A. Differences in cardiac and respiratory deterioration among group A and group B are intriguing and are in line with previous observations by Semplicini et al. which reported earlier respiratory involvement in severe patients compared to milder ones (18) and also with the recent demonstration of a tight connection between cardiac and respiratory manifestations and disease duration (13). Little is known on the functional role of β-sg in cardiac and lung tissues. Mutations in the β-sg gene might affect different tissue-specific pathways leading to diverse declines of muscle, cardiac, and respiratory functions. Therefore, an understanding of the relationships between genotype and phenotype in group A is important for informing diagnosis and disease management, as well as the development of genetic therapies. Although western blot analyses of β-sg were not available in participants, the severity of the cardiorespiratory progression might be related to the complete or severe loss of β-sg expression caused by the truncated gene mutation of group A patients (13). As protein expression higher than 30% has been related to a milder phenotype (13), gene replacement could be a valuable tool especially in these severely affected patients, as it could enable to change the disease progression and eventually prevent systemic involvement (25).

The number of clinical data and evaluations was higher in group A than in group B, thus limiting our comparisons. This is likely due to the milder clinical presentation of group B patients, requiring a less close follow-up routine. Limitations of this study include the small sample size determined by the rarity of LGMDR4 and differences in follow-up routines between clinical sites. Moreover, in the case of muscle function involvement, we only collected data regarding age of losing ambulation. Consequently, our results must be treated with caution and will require more future detailed statistical analyses with high number of patients to corroborate the reliability of our observations. On the other hand, studies looking at clinically relevant endpoints of the disease progression could select more advanced LGMDR4 patients to determine the effectiveness of the therapy.

## Conclusions

We studied the evolution over time of common variables that can be measured multiple times over the course of a structured follow-up for LGMDR4. Both respiratory and cardiac functions show a significant decrease over the years. The worsening of muscle function is faithfully described by the decrease in CPK, which has proven to be the simplest and least expensive parameter not only to closely follow the disease progression but also to predict clinical outcomes, such as the need to introduce respiratory support. Among respiratory variables, VC, FVC, and FEV1 were significantly correlated with age, suggesting a decrease in respiratory muscle compliance (13, 18). In addition, EF was the biomarker that most steadily decreases with aging and disease progression. Finally, we confirmed the existence of a phenotype–genotype correlation, noting a considerably more severe muscle damage and therefore a worse disease course in patients with the truncating mutation c.377_384duplCAGTAGGA on exon 3, both in homozygosis and heterozygosis.

## Data Availability Statement

The original contributions presented in the study are included in the article/Supplementary Material, further inquiries can be directed to the corresponding author/s.

## Ethics Statement

The studies involving human participants were reviewed and approved by the Ethics Committee of Policlinico of Milano, Italy. Written informed consent was collected from the patient for the inclusion of deidentified clinical data in a scientific publication, in accordance with the Declaration of Helsinki. Written informed consent to participate in this study was provided by the participants' legal guardian/next of kin.

## Author Contributions

GBM: investigation and writing original drafts. LV: supervision, statistical analysis, and writing review and editing. YT: conceptualization, supervision, and writing review and editing. All authors contributed to the article and approved the submitted version.

## Conflict of Interest

The authors declare that the research was conducted in the absence of any commercial or financial relationships that could be construed as a potential conflict of interest.

## Supplementary Material

Supplementary Table 1 Association between the rate in evolution of disease biomarkers and age at development of clinically relevant outcomes, disability, as evaluated by generalized linear models. CPK, Creatine Phosphokinase; EF, ejection fraction; VC, Vital Capacity, considered as percentage of predicted; FVC, Forced Vital Capacity, considered as percentage of predicted; FEV1, Forced Expiratory Volume in the first second considered as percentage of predicted. *N* = number of observations recorded.
