# Supplementary material for: Clinical Determinants of Disease Progression in Patients With Beta-Sarcoglycan Gene Mutations
Source: Front Neurol. 2021 Jul 1;12:657949. doi: 10.3389/fneur.2021.657949 (PMC8280524; doi:10.3389/fneur.2021.657949)
Supplement: Supplementary Table 1 — Association between the rate in evolution of disease biomarkers and age at development of clinically relevant outcomes, disability, as evaluated by generalized linear models. CPK, Creatine Phosphokinase; EF, ejection fraction; VC, Vital Capacity, considered as percentage of predicted; FVC, Forced Vital Capacity, considered as percentage of predicted; FEV1, Forced Expiratory Volume in the first second considered as percentage of predicted. N = number of observations recorded. [file Table_1.docx]

|  | **AGE AT LOSS OF AMBULATION** | | | | **AGE AT INTRODUCTION OF CPAP/COUGH ASSIST/AMBU** | | | | **AGE AT INTRODUCTION OF CARDIAC SUPPORT THERAPY** | | | | |
| --- | --- | --- | --- | --- | --- | --- | --- | --- | --- | --- | --- | --- | --- |
|  | **N** | **Estimate** | **Std Error** | **P value** | **N** | **Estimate** | **Std Error** | **P value** | **N** | **Estimate** | **Std Error** | **P value** |  |
| CPK IU/l/ year | 13 | -4,35E-04 | 4,22E-03 | 9,20E-01 | 10 | 1,67E-02 | 3,28E-03 | 0,0009* | 10 | 6,08E-03 | 6,60E-03 | 3,84E-01 |  |
| EF, %/year | 10 | -8,35E-01 | 3,15E+00 | 7,97E-01 | 6 | -3,42E+00 | 3,89E+00 | 4,30E-01 | 9 | -1,68E+00 | 3,04E+00 | 5,97E-01 |  |
| VC, %/year | 6 | -7,58E-02 | 2,84E-01 | 8,02E-01 | 5 | -2,07E+00 | 1,54E+00 | 2,71E-01 | 5 | -1,25E+00 | 7,07E-01 | 1,76E-01 |  |
| FVC, %/year | 8 | 8,75E-01 | 4,13E-01 | 7,83E-02 | 7 | 1,12E+00 | 2,05E+00 | 6,07E-01 | 5 | 1,30E+00 | 1,92E+00 | 5,48E-01 |  |
| FEV1, %/year | 10 | 3,49E-01 | 3,18E-01 | 3,05E-01 | 8 | 3,51E-01 | 1,25E+00 | 7,89E-01 | 7 | -1,41E-01 | 1,11E+00 | 9,04E-01 |  |

Supplementary table 1 (S1)

*Abbreviations:*

CPK= Creatine Phosphokinase, EF= ejection fraction, VC= Vital Capacity, considered as percentage of predicted, FVC= Forced Vital Capacity, considered as percentage of predicted, FEV1= Forced Expiratory Volume in the first second considered as percentage of predicted.

N = number of observations recorded.
